# Gout and the risk of age-related macular degeneration in the elderly

**DOI:** 10.1371/journal.pone.0199562

**Published:** 2018-07-12

**Authors:** Jasvinder A. Singh, John D. Cleveland

**Affiliations:** 1 Medicine Service, VA Medical Center, Birmingham, Alabama, United States of America; 2 Department of Medicine at the School of Medicine, University of Alabama at Birmingham (UAB), Birmingham, Alabama, United States of America; 3 Department of Epidemiology at the UAB School of Public Health, Birmingham, Alabama, United States of America; National Yang-Ming University Hospital, TAIWAN

## Abstract

**Objective:**

To assess whether gout is associated with incident age-related macular degeneration (AMD)

**Methods:**

We used the 5% Medicare claims data from 2006–12 for all beneficiaries who were enrolled in Medicare fee-for-service (Parts A, B) and not enrolled in a Medicare Advantage Plan, and resided in the U.S. People were censored at the occurrence of new diagnosis of AMD, death or the end of study (12/31/2012), whichever occurred first. We used multivariable-adjusted Cox regression analyses to assess the association of gout with incident AMD, adjusted for demographics, comorbidity, and use of medications for cardiovascular disease and gout. Hazard ratios and 95% confidence intervals were calculated.

**Results:**

In this observational cohort study, of the 1,684,314 eligible people, 116,097 developed incident AMD (6.9%). Incidence rates of AMD per 1,000 person-years were 20.1 for people with gout and 11.7 for people without gout. In multivariable-adjusted analyses, a diagnosis of gout was significantly associated with a higher risk of incident AMD with a hazard ratio of 1.39 (95% CI, 1.35, 1.43). This association was confirmed in sensitivity analyses that substituted Charlson-Romano comorbidity index continuous score with either a categorical Charlson-Romano comorbidity index score or individual Charlson-Romano index comorbidities plus hypertension, hyperlipidemia and coronary artery disease. Other covariates significantly associated with higher hazards of incident AMD were older age, female gender, White race/ethnicity, and higher Charlson-Romano comorbidity index score.

**Conclusions:**

We noted a novel association of gout with AMD in the elderly. Future studies should investigate the pathways that mediate this association.

## Introduction

Age-related macular degeneration (AMD) is a common eye condition and a leading cause of blindness in adults 50 years or older in developed countries [1]. Approximately 8 million Americans have AMD [2]. AMD is characterized by extensive drusen, often associated with pigmentary abnormalities. Due to damage to macula near the center of the retina, which is needed for sharp central vision, patients with AMD have vision loss in one or both eyes and/or a blurred area near the center of vision. AMD is associated with significant morbidity due to its effect on central vision and a higher mortality [3–5]. Age, smoking, Caucasian race/ethnicity and a family history of AMD are risk factors for AMD [6, 7]. The exact mechanisms for AMD are unclear.

Recent studies provide evidence of a link between inflammation and the pathogenesis of AMD [8–16]. While no association was noted between arthritis and AMD in two cross-sectional studies [14] [17], a prospective study showed that people with rheumatoid arthritis had significantly increased risk of AMD, with a rate ratio of 1.15 [18]. Oxidative stress, caused by excessive production of reactive oxygen species and body’s ability to readily detoxify the reactive intermediates or to repair the resulting damage,[19] might also play an important role in AMD. The use of antioxidants (vitamins and zinc) can reduce the risk of developing advanced AMD and intravitreal treatment with ranibizumab, a vascular endothelial growth factor (VEGF) monoclonal antibody, reduces or improves loss of vision in AMD [20]. Therefore, it is possible that other conditions associated with oxidative stress and inflammation, such as rheumatic diseases such as rheumatoid arthritis and others, cancer, and cardiovascular disease,[21] can increase the risk of AMD.

Gout, the most common inflammatory arthritis affecting 3.9% U.S. adults, is associated with oxidative stress [22, 23], that is partially related to hyperuricemia, a cardinal feature of gout [24]. Disease epidemiology is similar; gout prevalence increases with age with a significant increase after age 65 and especially after age 75, [25] similar to AMD. Adults 65 years of older is an important population to study, since their number will increase from 34.4 million in 2000 to more than 70 million in 2030 in the U.S. [26]. Studies of AMD risk factors in this population are needed to improve our understanding of the burden of AMD and offer potential insights into disease mechanisms.

Our study objective was to address two important questions in this population, namely, whether gout increases the risk of AMD in the older adults, and if this varies by gender, and race/ethnicity.

## Materials and methods

### Data sources and data abstracted

This retrospective cohort study used the 5% Medicare claims sample obtained from the Centers for Medicare and Medicaid Services (CMS) Chronic Condition Data Warehouse. Medicare is a federal health insurance program that pays for a variety of health care expenses, administered by CMS, a division of the U.S. Department of Health & Human Services (HHS) that covers 59 million Americans currently. Medicare beneficiaries are typically senior citizens aged 65 and older, although adults with qualifying permanent disabilities or certain approved medical conditions (such as end-stage renal disease or Lou Gehrig’s disease) may also be eligible for Medicare benefits.[27] Thus, U.S. Medicare cohort is representative of U.S. adults 65 years or older. We included all insurance claims for each beneficiary. We abstracted all prescription claims (dose, supply, and drug name) from Medicare part D file, all inpatient and outpatient claim files including diagnosis codes and claim dates from Medicare part A and B files, and demographics (birthdate, death date, sex, and race) from the beneficiary summary file for each beneficiary.

Study inclusion criteria were as follows: (1) Medicare beneficiaries who were enrolled in Medicare fee-for-service (Parts A, B) and not enrolled in a Medicare Advantage Plan (part C) during the period 2006–2012 (Medicare part C were excluded since these patients have incomplete medication claims); and (2) resided in the U.S. during the study period (2006–2012). The Institutional Review Board (IRB) at the University of Alabama at Birmingham (UAB) approved the study and waived the requirement for Informed consent for this study of analyses of claims data retrospectively. We reported study methods and results in accordance with the Strengthening of Reporting in Observational studies in Epidemiology (STROBE) statement [28]. People were censored at the occurrence of new diagnosis of AMD, death or the end of the study, i.e., 12/31/2012, whichever occurred first. The University of Alabama at Birmingham’s Institutional Review Board approved this study and waived the need for informed consent for this database study. All investigations were conducted in conformity with ethical principles of research.

### Study outcome

The study outcome was the development of incident AMD, identified by the new occurrence of an International Classification of Diseases, ninth revision (ICD-9) diagnostic codes 362.50, 362.51 or 362.52, with an absence of this diagnosis in the baseline period of 365 days (1/1/2005 to 12/31/2005). This algorithm to identify AMD was based on a validated algorithm previously used with both specificity and positive predictive values exceeding 95% [29]. Thus, the study follow-up period was from 2006–2012, with exclusion of people who had AMD in 2006.

### Predictor of interest

The predictor of interest was gout, diagnosed before AMD. We identified gout by the presence of two ICD-9-CM diagnostic codes, 274.xx, in the claims data. Patients met the definition on the date of the occurrence of the second diagnostic code for gout. ICD-9 codes for gout have high accuracy with sensitivity of 90% and specificity of 100% [30].

### Covariates and confounders

We assessed important covariates and confounders for our outcome of interest, which included patient demographics and comorbidity in the baseline period, and the use of common medications for cardiovascular disease and for gout, all obtained from the Medicare denominator file and claims data. Demographics included age, gender and race/ethnicity. We assessed medical comorbidity using the Charlson-Romano index, a commonly used validated comorbidity index that is calculated using the claims data [31]. It is a weighted comorbidity index consisting of comorbidities such as myocardial infarction, heart failure, cerebrovascular disease, liver disease, pulmonary disease, renal disease, peripheral vascular disease, etc. [32]. We included medications commonly used for the treatment of cardiovascular diseases, namely, statins, beta-blockers, diuretics, and angiotensin converting enzyme (ACE)-inhibitors. We also included commonly used urate-lowering medications for gout, allopurinol and febuxostat. All medications were current use, and we examined them as time-varying covariates. We included these medications to account for presence of common cardiac conditions and gout (where diagnosis may have been delayed), as an imperfect surrogate for severity of these conditions in the absence of disease severity measures in Medicare claims data, and their potential positive impact on oxidative stress, [33–35] a potential pathway for AMD.

### Statistical analyses

To check the balance of key characteristics and identify possible confounders in this large observational study, we compared characteristics of patients who did vs. did not develop incident AMD during the follow-up using a t-test or a chi-square test, as appropriate. We used a series of multivariable-adjusted Cox proportional hazard models to assess the independent association of gout with incident (new) AMD, adjusted for the potentially imbalanced characteristics: age (65—<75, 75-<85, ≥85 years), gender (male, female), race (White, Black, Other), Charlson-Romano comorbidity score (continuous score variable), cardiovascular medications (statins, beta-blockers, diuretics, ACE-inhibitors, categorized as current use vs. non-use) and gout medications (allopurinol, febuxostat categorized as current use vs. non-use; main model; model 1). Hazard ratios (HR) with 95% confidence intervals were estimated. Since comorbidities are the main competing/potential confounders, we ran three models with varying ways of examining medical comorbidities as sensitivity analyses to test robustness of our results: (1) the Charlson-Romano score as a continuous variable (model 1); (2) the Charlson-Romano score as a categorical variable (score of 0, 1 or ≥2; model 2); and (3) using individual Charlson-Romano score comorbidities, hypertension, hyperlipidemia and coronary artery disease (presence vs. absence of each comorbidity; model 3). We used SAS version 9.4 (Cary, NC) to conduct the analyses.

## Results

### Characteristics of the study population and crude incidence rate of AMD

**Fig 1:**
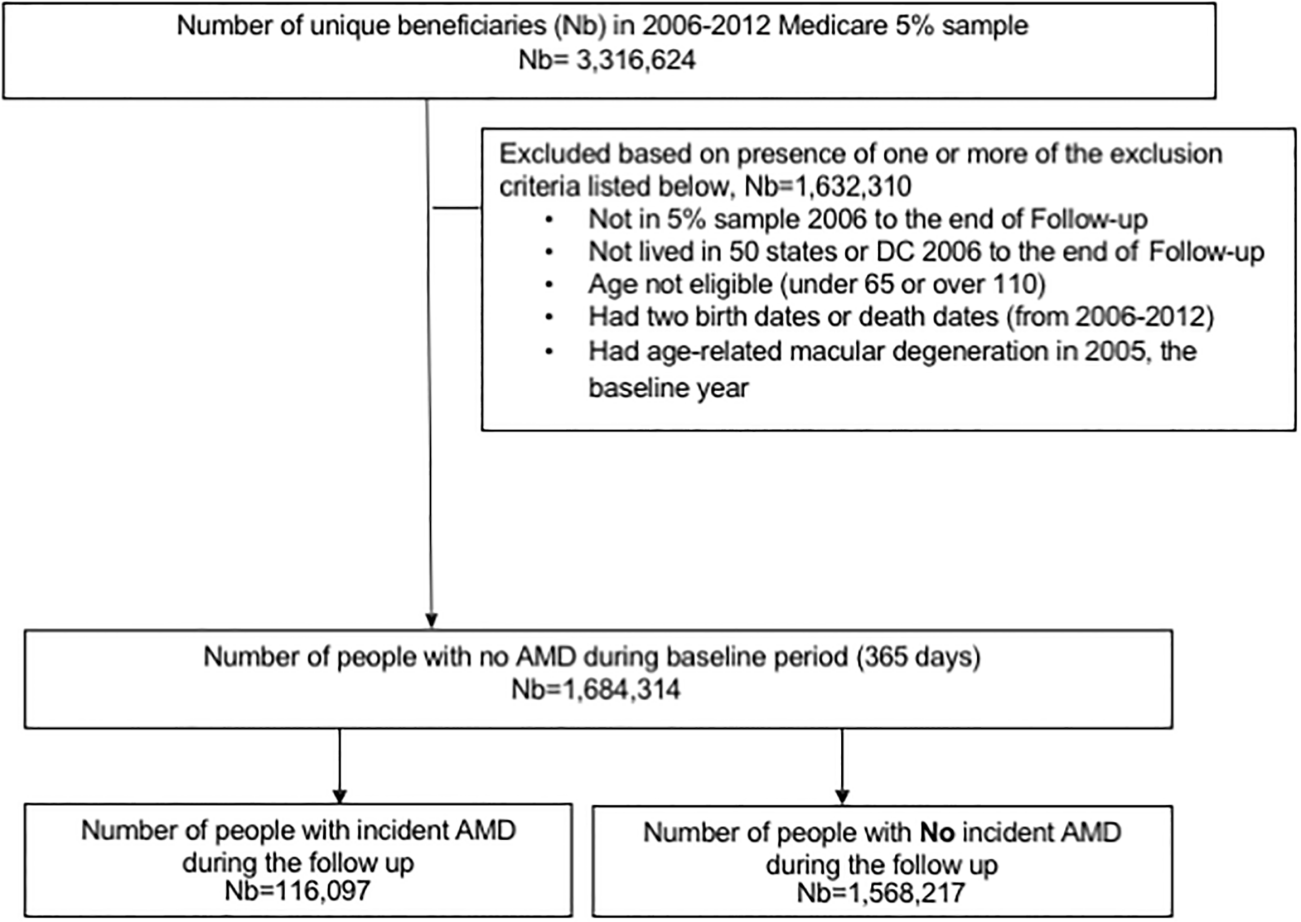
Study cohort flow chart. Age-related macular degeneration; Nb, number of unique beneficiaries.

Of the 1,684,314 eligible people, 116,097 (6.9%) developed incident AMD (Fig 1). Compared to people who did not develop AMD, those who developed AMD were older, more likely to be female, or White; had higher Charlson-Romano comorbidity index score, or were likely to have hypertension, hypertension or coronary artery disease (Table 1).

**Table 1:** Demographic and clinical characteristics of episodes of age-related macular degeneration (AMD).

	All episodes	Macular Degeneration during the follow-up		p-value
		No	Yes	
Total, N (episodes)	1,684,314*	1,568,217	116,097	
Age, mean (SD)	75.2 (7.5)	**75.0 (7.5)**	**77.4 (7.1)**	**<0.0001**
Gender, N (%)				**<0.0001**
Male	719,472 (42.7%)	**677,962 (43.2%)**	**41,510 (35.8%)**	
Female	964,842 (57.3%)	**890,255 (56.8%)**	**74,587 (64.2%)**	
Race/Ethnicity, N (%)				**<0.0001**
White	1,445,930 (85.8%)	**1,337,916 (85.3%)**	**108,014 (93.0%)**	
Black	141,866 (8.4%)	**138,873 (8.9%)**	**2,993 (2.6%)**	
Other/unknown	96,518 (5.7%)	**91,428 (5.8%)**	**5,090 (4.4%)**	
Charlson				**<0.0001**
0	893,385 (53.0%)	**843,496 (53.8%)**	**49,889 (43.0%)**	
1	167,884 (10.0%)	**153,336 (9.8%)**	**14,548 (12.5%)**	
≥2	623,045 (37.0%)	**571,385 (36.4%)**	**51,660 (44.5%)**	
Charlson-Romano comorbidity score, mean (SD)	1.59 (2.40)	**1.58 (2.41)**	**1.77 (2.24)**	**<0.0001**
Charlson-Romano comorbidities				
Myocardial Infarction	66,515 (3.9%)	**61,369 (3.9%)**	**5,146 (4.4%)**	**<0.0001**
Heart Failure	196,190 (11.6%)	**181,100 (11.5%)**	**15,090 (13.0%)**	**<0.0001**
Peripheral vascular disease	162,521 (9.6%)	**148,343 (9.5%)**	**14,178 (12.2%)**	**<0.0001**
Cerebrovascular disease	162,289 (9.6%)	**148,242 (9.5%)**	**14,047 (12.1%)**	**<0.0001**
Dementia	76,347 (4.5%)	**72,535 (4.6%)**	**3,812 (3.3%)**	**<0.0001**
Chronic pulmonary disease	260,960 (15.5%)	**239,072 (15.2%)**	**21,888 (18.9%)**	**<0.0001**
Connective tissue disease	46,036 (2.7%)	**41,543 (2.6%)**	**4,493 (3.9%)**	**<0.0001**
Peptic ulcer disease	31,543 (1.9%)	**28,691 (1.8%)**	**2,852 (2.5%)**	**<0.0001**
Mild liver disease	8,300 (0.49%)	7,723 (0.49%)	577 (0.50%)	0.8316
Diabetes	310,802 (18.5%)	**287,109 (18.3%)**	**23,693 (20.4%)**	**<0.0001**
Diabetes with end organ damage	91,815 (5.5%)	**84,931 (5.4%)**	**6,884 (5.9%)**	**<0.0001**
Hemiplegia	13,990 (0.83%)	**13,108 (0.84%)**	**882 (0.76%)**	**0.0058**
Renal failure/disease	57,729 (3.4%)	**53,624 (3.4%)**	**4,105 (3.5%)**	**0.0354**
Any tumor leukemia lymphoma	167,639 (10.0%)	**153,012 (9.8%)**	**14,627 (12.6%)**	**<0.0001**
Moderate or severe liver disease	1,958 (0.12%)	**1,861 (0.12%)**	**97 (0.08%)**	**0.0007**
Metastatic cancer	17,567 (1.0%)	**16,671 (1.1%)**	**896 (0.77%)**	**<0.0001**
AIDS	550 (0.03%)	**527 (0.03%)**	**23 (0.02%)**	**0.0121**
Gout	87,524 (5.2%)	81,963 (5.2%)	5,561 (4.8%)	**<0.0001**
Hypertension	804,211 (47.7%)	**732,198 (46.7%)**	**72,013 (62.0%)**	**<0.0001**
Hyperlipidemia	579,951 (34.4%)	**526,408 (33.6%)**	**53,543 (46.1%)**	**<0.0001**
Coronary artery disease	292,716 (17.4%)	**266,224 (17.0%)**	**26,492 (22.8%)**	**<0.0001**

*met eligibility criteria and did not have AMD in the baseline 365-day period

Incidence rates of AMD per 1,000 person-years were 20.1 for people with gout and 11.7 for people without gout (Table 2).

**Table 2:** Crude incidence rate of age-related macular degeneration (AMD) by the presence of gout at baseline.

	Person-months of follow up*	Person years	#Cases of Macular Degeneration	Macular Degeneration Incidence Rate per 100,000 person-months	Macular Degeneration incidence rate per 1,000 person-years (95% CI)
Gout	3,318,661	276,555	5,561	167.6	20.1 (19.6, 20.6)
No Gout	113,009,569	9,417,464	110,536	97.8	11.7 (11.7, 11.8)

*Follow-up continued until the occurrence of new diagnosis of AMD, death or end of study, 12/31/2012, whichever occurred first, at which point observations were censored

### Multivariable adjusted association of gout and AMD

In the main model adjusted for Charlson comorbidity index score, gout was significantly associated with a higher risk of incident AMD, hazard ratio was 1.39 (95% CI, 1.35, 1.43) (Table 3). Other covariates significantly associated with higher hazards of incident AMD were older age, female gender, White race, and a higher Charlson-Romano comorbidity index score (Table 3).

**Table 3:** Association of gout and other risk factors with age-related macular degeneration (AMD).

	Multivariable-adjusted (Model 1)		Multivariable-adjusted (Model 2)		Multivariable-adjusted (Model 3)	
	HR (95% CI)	P-value	HR (95% CI)	P-value	HR (95% CI)	P-value
Age (in years)						
65—<75	Ref		Ref		Ref	
75—<85	**2.18 (2.16, 2.21)**	**<0.0001**	**2.14 (2.12, 2.17)**	**<0.0001**	**2.10 (2.07, 2.13)**	**<0.0001**
≥85	**3.23 (3.17, 3.28)**	**<0.0001**	**3.17 (3.11, 3.22)**	**<0.0001**	**3.24 (3.18, 3.30)**	**<0.0001**
Gender						
Male	Ref		Ref		Ref	
Female	**1.26 (1.25, 1.28)**	**<0.0001**	**1.26 (1.25, 1.28)**	**<0.0001**	**1.25 (1.23, 1.26)**	**<0.0001**
Race						
White	Ref		Ref		Ref	
Black	**0.27 (0.26, 0.28)**	**<0.0001**	**0.27 (0.26, 0.28)**	**<0.0001**	**0.28 (0.27, 0.29)**	**<0.0001**
Other	**0.66 (0.64, 0.68)**	**<0.0001**	**0.67 (0.65, 0.69)**	**<0.0001**	**0.69 (0.67, 0.71)**	**<0.0001**
Charlson-Romano score, per unit change	**1.09 (1.09, 1.10)**	**<0.0001**				
Gout	**1.39 (1.35, 1.43)**	**<0.0001**	**1.36 (1.33, 1.40)**	**<0.0001**	**1.25 (1.22, 1.29)**	**<0.0001**
Statins	**0.94 (0.92, 0.95)**	**<0.0001**	**0.93 (0.92, 0.95)**	**<0.0001**	**0.90 (0.88, 0.91)**	**<0.0001**
Beta blockers	1.02 (1.00, 1.03)	0.10	1.02 (1.00, 1.04)	0.028	0.99 (0.97, 1.01)	0.34
Diuretics	0.99 (0.97, 1.00)	0.13	0.99 (0.97, 1.01)	0.18	**0.96 (0.95, 0.98)**	**<0.0001**
ACE inhibitor	**0.89 (0.87, 0.90)**	**<0.0001**	**0.88 (0.86, 0.90)**	**<0.0001**	**0.88 (0.86, 0.90)**	**<0.0001**
Allopurinol	**0.89 (0.84, 0.93)**	**<0.0001**	**0.91 (0.86, 0.95)**	**0.0001**	**0.93 (0.88, 0.98)**	**0.004**
Febuxostat	1.18 (0.85, 1.64)	0.33	1.19 (0.85, 1.65)	0.31	1.20 (0.86, 1.67)	0.29
Charlson- Romano = 0						
0						
1			Ref			
≥2			**1.56 (1.53, 1.59)**	**<0.0001**		
			**1.74 (1.72, 1.76)**	**<0.0001**		
Myocardial Infarction					**0.91 (0.88, 0.93)**	**<0.0001**
Heart Failure					**1.06 (1.04, 1.08)**	**<0.0001**
Peripheral vascular disease					**1.16 (1.14, 1.18)**	**<0.0001**
Cerebrovascular disease					**1.11 (1.08, 1.13)**	**<0.0001**
Dementia					**0.83 (0.80, 0.85)**	**<0.0001**
Chronic pulmonary disease					**1.24 (1.22, 1.26)**	**<0.0001**
Connective tissue disease					**1.24 (1.20, 1.27)**	**<0.0001**
Peptic ulcer disease					**1.16 (1.12, 1.20)**	**<0.0001**
Mild liver disease					**1.16 (1.06, 1.26)**	**0.001**
Diabetes					1.01 (1.00, 1.03)	0.14
Diabetes with end organ damage					**1.07 (1.04, 1.10)**	**<0.0001**
Hemiplegia					**0.94 (0.87, 1.00)**	**0.049**
Renal failure/disease					**1.03 (1.00, 1.07)**	**0.044**
Any tumor leukemia lymphoma					**1.27 (1.25, 1.30)**	**<0.0001**
Moderate or severe liver disease					1.04 (0.84, 1.27)	0.74
Metastatic cancer					0.99 (0.92, 1.06)	0.71
AIDS					1.06 (0.71, 1.60)	0.78
Hypertension					**1.48 (1.46, 1.51)**	**<0.0001**
Hyperlipidemia					**1.20 (1.18, 1.22)**	**<0.0001**
Coronary artery disease					**1.14 (1.12, 1.16)**	**<0.0001**

HR, Hazard ratio; CI, confidence interval; Ref, referent category

All models were performed with death as a competing risk

In sensitivity analyses that either adjusted for Charlson-Romano comorbidity index as a categorical variable (model 2) or for individual Charlson-Romano score comorbidities (model 3), gout was significantly associated with incident AMD; respective hazard ratios were 1.36 (95% CI, 1.33, 1.40) and 1.25 (95% CI, 1.22, 1.29) (Table 3). Most previously associations were still evident and were minimally attenuated (Table 3).

In a sensitivity multivariable-adjusted analyses for Model 1, we limited the Medicare sample to those that survived to the end of the study period, i.e., 12/31/18 (n = 1,201,090), the association of gout with incident AMD was essentially the same, the HR was 1.40 (95% CI, 1.35, 1.44).

**Fig 2:**
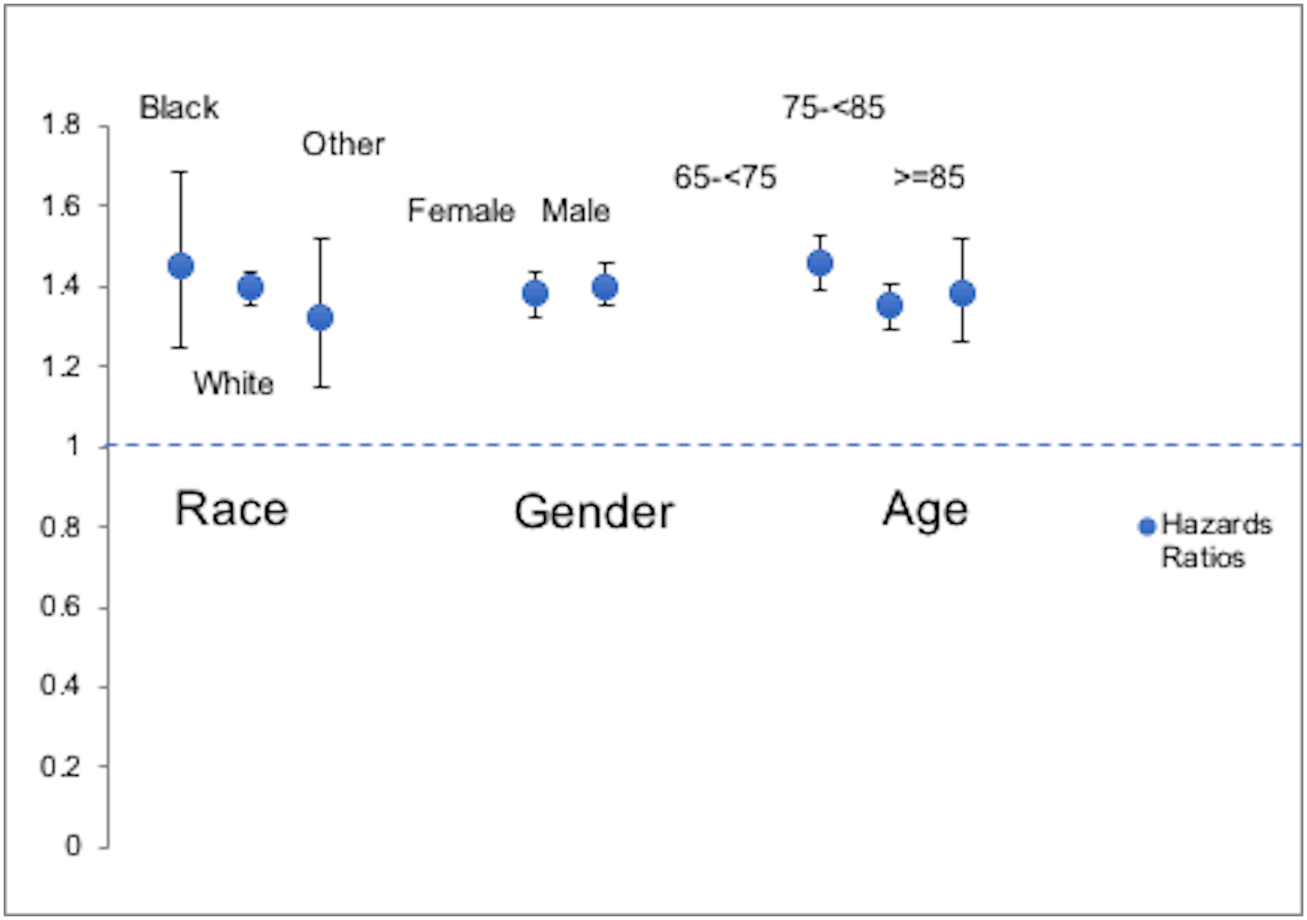
Subgroup analyses of association of gout with incident AMD by demographics. Point estimates indicate hazard ratios and the whiskers represent the 95% confidence intervals (CI).

Subgroup analyses by race, gender showed no significant interaction with the association of gout with AMD, but age showed a significant interaction with this association, with highest increase in hazards of AMD with gout in the age group 65 to <75 years (Fig 2 and S1 Table).

## Discussion

In this study of elderly people 65 years and older, who were Medicare beneficiaries, we found that gout was an independent risk factor for the development of AMD. Higher medical comorbidity was associated with higher hazard/risk of AMD, as were most of the Charlson-Romano score comorbidities, hypertension, hyperlipidemia, and coronary artery disease. We confirmed previously observed associations of older age and White race with a higher risk of AMD.[6, 7] A higher risk of AMD in Whites may be due to social and/or biological factors.

A novel study finding was that in this large observational study, gout was an independent risk factor for the development of AMD, even after controlling for a number of potential confounders. Gout increased the hazard of incident AMD by 40% in the main model. A possible explanation is that gout is associated with hyperuricemia,[36, 37] which is associated with increased oxidative stress [22, 38, 39] and urate-crystal associated inflammation.[40, 41] Oxidative stress is as an important pathway for AMD.[11, 15, 42–47] Treatment with antioxidants (vitamins and zinc) reduced the risk of developing advanced AMD and treatment with ranibizumab, a VEGF monoclonal antibody, reduced the loss of vision, or in some cases improved vision in AMD.[20] Therefore, oxidative stress and systemic inflammation pathways are potential mechanisms for the higher AMD risk with gout in the elderly. There are several potential implications of this finding.

First, could the use of anti-oxidants such as Vitamin C, Vitamin E, selenium, beta-carotene delay or reduce the risk of AMD in patients with gout? Anti-oxidants had a beneficial effect compared to placebo in AMD patients [48, 49] and it is possible that in conditions such as gout where oxidative stress is higher than in general population, this benefit may even be greater. Patients with gout frequently use cherries and other health foods and supplements with anti-oxidant properties to reduce the risk of gout flares and this is an area of active research, rated a high priority by patients.[50, 51] A randomized study comparing a potentially effective anti-oxidant (or a combination) for AMD prevention in people with gout can answer this practical question. Another question is whether effective treatment of gout and achievement of disease remission, with minimization of flares and normalization of serum urate, can reduce the risk of AMD? A prospective cohort study or a randomized study with a longer follow-up can address this question. Comparison with historical controls and usual care, which frequently fails to achieve disease remission can also address this question. At the very least, the recognition of a common chronic inflammatory condition such gout as a potential risk factor for AMD is important. Our study does not suggest causation, only an association. A discussion related to this risk with elderly people with gout during regular clinic visits may help inform people about this risk.

Another new finding was the association of higher comorbidity with the risk of AMD. Compared to no medical comorbidity, presence of one or two or more medical comorbidities increased the hazard of AMD by 60% and 80% respectively. The mechanism of increased risk of AMD with higher comorbidity is unclear. Most likely candidate mechanisms are systemic inflammation, oxidative stress and endothelial dysfunction, which are features of several chronic conditions that we noted to be associated such as pulmonary disease, connective tissue disease, cancer etc. Another interesting observation was that hypertension was associated with a 48% increased hazard of AMD. Previous studies reported the association of hypertension with AMD [52–54], although some epidemiological studies failed to document this association [55, 56]. Our study extends the association of hypertension with AMD to a generalizable elderly U.S. cohort of 65 years or older.

Several comorbidities, including diseases that are manifestations of atherosclerotic disease (coronary artery disease, cerebrovascular disease, peripheral vascular disease, hyperlipidemia and diabetes) were associated with an increased risk of AMD. Our study was not focused on this hypothesis, yet these findings are interesting and hypothesis-generating. Data related to the association of atherosclerosis and AMD is conflicting with some previous epidemiological studies showing a positive association[57, 58] and others showing no such association [55, 56, 59]. Both positive and negative studies controlled for confounding bias, therefore, the reasons for differences in findings are unclear. The hazard ratios for AMD associated with hypertension and coronary artery disease in our study were similar to one previous study [58]. Given the conflicting data in this area, more data from high-quality observational studies are needed in the future to clarify whether atherosclerotic diseases are modifiable risk factors for AMD or not.

Our study has several limitations and strengths. Our findings can only be generalized to elderly 65 years or older. Misclassification bias is another potential limitation of our observational cohort study that relied on diagnostic codes. Even though algorithms to identify gout [30] and AMD [29] have been shown to have high accuracy, we can not rule out a possibility of misclassification. Confounding bias, a common issue in observational studies is another limitation. We used Cox regression model and covariance adjustment by including several imbalanced covariates in our multivariable-adjusted model to account for their effects, and performed several sensitivity analyses, that confirmed the robustness of our findings. We were unable to control for body mass index, smoking status, socioeconomic status or education level, which were not available in the Medicare claims. However, alternative adjusting methods such as structural/marginal model, G-estimation causal analysis methods, stratification and matching methods, instrumental variable techniques, propensity models and other methods might be also implemented. We did not perform additional analyses including death as a competing cause in Cox regression analyses[60] due to limited resources, which could have provided additional insight into whether gout’s association with AMD would vary with one more sensitivity analysis model. However, analyses limited to a subsample of beneficiaries that survived to 12/31/2012 essentially reproduced the same HR as the main model 1, and subgroup analyses were performed by age, race and gender for assessing the competing risk in a proportional hazard model.

Although gout is not a previously known risk factor for AMD and would not be associated with surveillance bias for AMD, a higher comorbidity load in people with gout may have led to higher rate of surveillance for AMD compared to people with gout. On the other hand, a longer mean follow-up observation period for non-gout vs. gout patients (5.9 vs. 3.1 years) may have increased detection bias in the non-gout population, making our estimates conservative. Therefore, given both positive and negative factors, we can not estimate the overall direction or magnitude of the surveillance or detection bias in our study, which may have partially contributed to the noted association. In some cases, patients with gout may suffer from prior macular vascular occlusions and this may be interpreted, by some physicians, as non-exudative macular degeneration; this diagnostic error could have some impact on the noted association, although we do not believe that diagnostic error could explain the association completely.

Strengths of our study include the use of a representative sample, a large sample size, adjustment for several important covariates, use of validated algorithms for identification of AMD and gout and replication of results in sensitivity analyses.

### Conclusions

In conclusion, we found that gout was association with a higher risk of AMD in adults 65 years and older. Other independent risk factors for AMD in this patient population were female gender, older age, White race and higher comorbidity. Future studies should investigate the pathways that put patients with gout at higher risk of AMD. Randomized trials are also needed to assess whether the use of anti-oxidants in this patient population can simultaneously reduce the risk of AMD and of gout disease activity.

## Supporting information

S1 Table Association of gout with AMD, in pre-defined subgroup analyses, by race, gender, and age. gout*race p-value 0.1125; Gout*gender p-value 0.1441; Age*gout p-value 0.0137. HR, Hazard ratio; CI, confidence interval; Hazard ratios that are significant with p-value <0.05 are in bold.
(DOCX)

## Abbreviations

ACE inhibitor: Angiotensin converting enzyme inhibitor
CAD: coronary artery disease
CMS: Centers for Medicare and Medicaid Services
ICD-9-CM: International Classification of Diseases, ninth revision, common modification
ULT: urate-lowering therapy
