# Supplementary material for: Gout and the risk of age-related macular degeneration in the elderly
Source: PLoS One. 2018 Jul 12;13(7):e0199562. doi: 10.1371/journal.pone.0199562 (PMC6042699; doi:10.1371/journal.pone.0199562)
Supplement: S1 Table — gout*race p-value 0.1125; Gout*gender p-value 0.1441; Age*gout p-value 0.0137. HR, Hazard ratio; CI, confidence interval; Hazard ratios that are significant with p-value <0.05 are in bold. (DOCX) [file pone.0199562.s001.docx]

Supplementary Table 1. Association of gout with AMD, in pre-defined subgroup analyses, by race, gender, and age

|  | Multivariable-adjusted (Model 1) | | Multivariable-adjusted  (Model 1) | | Multivariable-adjusted  (Model 1) | |
| --- | --- | --- | --- | --- | --- | --- |
|  | HR (95% CI) | P-value | HR (95% CI) | P-value | HR (95% CI) | P-value |
|  | **Black** | | **White** | | **Other race** | |
| Gout | **1.45 (1.25, 1.69)** | **<0.0001** | **1.40 (1.35, 1.44)** | **<0.0001** | **1.32 (1.15, 1.52)** | **0.0001** |
|  |  |  |  |  |  |  |
|  | **Female** | | **Male** | |  |  |
| Gout | **1.38 (1.32, 1.44)** | **<0.0001** | **1.40 (1.35, 1.46)** | **<0.0001** |  |  |
|  |  |  |  |  |  |  |
|  | **65-75 years** | | **75-85 years** | | **>85 years** | |
| Gout | **1.46 (1.39, 1.53)** | **<0.0001** | **1.35 (1.29, 1.41)** | **<0.0001** | **1.38 (1.26, 1.52)** | **<0.0001** |
|  |  |  |  |  |  |  |
|  |  |  |  |  |  |  |
| **gout*race p-value 0.1125; Gout*gender p-value 0.1441; Age*gout p-value 0.0137**  HR, Hazard ratio; CI, confidence interval;  Hazard ratios that are significant with p-value <0.05 are in bold | | | | | | |
